# Sleep disturbances in the ICU

**DOI:** 10.1186/cc9959

**Published:** 2011-03-11

**Authors:** G Langevoort, J Hofhuis, J Rommes, P Spronk

**Affiliations:** 1 Gelre Hospitals, Apeldoorn, the Netherlands

## Introduction

Sleep disturbances are common in critically ill patients on the ICU, with possibly serious consequences [1]. More attention is needed for the sleep-wake cycle of ICU patients. The aim of this study was to gain insight into factors that are important for sleep of critically ill patients on the ICU.

## Methods

We conducted a multicentre, exploratory survey sent to nurse managers of all adult ICUs in the Netherlands. We used a self-developed questionnaire to describe which factors are important for sleep of ICU patients. Surveys were distributed via mail with subsequent written reminders. Relevant factors in relation with sleep of ICU patients were included in the questionnaire.

## Results

The survey response rate was 60% (68/114). Characteristics of the sleeping patient on the ICU most often included: lying quiet with closed eyes (89.7%), decreased pulse rate (88.2%) and slower respiration (83.8%). Nonpharmacological interventions to improve sleep of the ICU patients most often comprised: keeping patients awake during the day (94.2%), lights out in the ICU (92.6%), use of a clock (91.2%), reducing noise of the ICU staff (89.7%) and reducing nursing interventions (86.8%). The type of sleep medication was mostly determined only by physicians (57.4%). The assessment of the effects of the sleep medication was mostly determined by nurses and physicians together (58.8%). Most frequent medications used were midazolam (92.6%), propofol (85.3%) and temazepam (75.1%). Nursing autonomy regarding sleep and sedation practices of patients (rated on a 10-point numerical scale) was judged as poor (median 5, IQR 3 to 7). How much nursing observations influences sleeping practices in the ICU was judged as good (median 8, IQR 7 to 8). How the average patient was sleeping on the ICU was judged as moderate (median 6, IQR 5 to 7). Furthermore, 69.1% of the ICUs mentioned a disturbed sleep-wake cycle, judged predominantly due to too much noise (61.8%), delirium (55.9%), and nursing interventions (48.5%). Most ICUs (83.8%) did not have a sleeping protocol, but 67.6% of these ICUs preferred to implement such a protocol.

## Conclusions

The average ICU patients are sleeping moderately well, mostly due to a disturbed sleep-wake cycle, delirium and nursing interventions. ICU nurses experience only a moderate feeling of autonomy and influence on sleeping practices. Most ICUs did not have a sleeping protocol, but more than one-half of these ICUs preferred to implement one.
